# Safety and Efficacy of Transarterial Chemoembolization in Elderly Patients with Intermediate Hepatocellular Carcinoma

**DOI:** 10.3390/cancers14071634

**Published:** 2022-03-23

**Authors:** Gael S. Roth, Olivier Hernandez, Najeh Daabek, Bleuenn Brusset, Yann Teyssier, Julien Ghelfi, Marie Noelle Hilleret, Christian Sengel, Ivan Bricault, Thomas Decaens, Charlotte Costentin

**Affiliations:** 1 Faculté de Médecine, Université Grenoble-Alpes, 38058 Grenoble, France; yteyssier@chu-grenoble.fr (Y.T.); jghelfi@chu-grenoble.fr (J.G.); ibricault@chu-grenoble.fr (I.B.); tdecaens@chu-grenoble.fr (T.D.); 2 Clinique Universitaire d’Hépato-Gastroentérologie et Oncologie Digestive, CHU Grenoble-Alpes, 38043 Grenoble, France; olivierhernandez2201@gmail.com (O.H.); bbrusset@chu-grenoble.fr (B.B.); mnhilleret@chu-grenoble.fr (M.N.H.); 3 Institute for Advanced Biosciences-INSERM U1209, CNRS UMR 5309, Université Grenoble-Alpes, 38042 Grenoble, France; 4 Laboratoire Hypoxie Physiopathologie (HP2) INSERM U1042M, Université Grenoble-Alpes, 38043 Grenoble, France; najeh.daabek1@gmail.com; 5 Biostatistiques, Action Contre la Faim, 75017 Paris, France; 6 Clinique Universitaire de Radiologie et Imagerie Médicale, Pôle Imagerie, CHU Grenoble-Alpes, 38043 Grenoble, France; csengel@chu-grenoble.fr

**Keywords:** intermediate hepatocellular carcinoma, transarterial chemoembolization, safety, elderly patients

## Abstract

**Simple Summary:**

Transarterial chemoembolization is the treatment of choice for intermediate hepatocellular carcinoma, which constitutes the second cause of cancer-related death worldwide. Even though the world population is aging, we lack evidence regarding the safety and efficacy of anticancer treatments in these patients. This study including 271 patients shows how elderly patients treated by transarterial chemoembolization have comparable safety profiles, tumor responses, and survival outcomes to younger patients. Transarterial chemoembolization should always be considered in elderly patients with good performance status and a compensated liver function presenting intermediate hepatocellular carcinoma.

**Abstract:**

(1) Introduction: Transarterial chemoembolization (TACE) is the most widely used treatment for intermediate hepatocellular carcinoma (HCC), with limited data available in elderly patients. This study compares the safety and efficacy of TACE for HCC in elderly patients (≥70 years) versus younger patients (<70 years). (2) Materials and Methods: Patients treated by a first TACE for HCC at Grenoble-Alpes University Hospital from January 2012 to March 2017 were included. The primary objective was to compare the safety and predictive factors of serious adverse events between groups using univariate and multivariate analyses. Secondary objectives included tumor response and survival analyses. (3) Results: 271 patients were included: 88 elderly and 183 under 70 years. A total of 20.5% of elderly patients experienced serious adverse events versus 21.3% of patients under 70 (*p* = 0.87). The predictive factors of serious adverse events were Child–Pugh ≥ B7 (*p* < 0.0001), ECOG ≥ 1 (*p* = 0.0019), and MELD ≥ 9 (*p* = 0.0415). The serious adverse event rate was not increased with age (*p* = 0.87). The objective tumor response rate was 89.5% in elderly versus 78.7% in younger patients (*p* = 0.03). (4) Conclusion: This study showed similar safety profiles of the first TACE between elderly and younger patients, with comparable efficacy outcomes, suggesting that advanced age should not constitute a limitation in itself in treatment decision-making.

## 1. Introduction

Liver cancer is the second cause of cancer-related deaths worldwide [1] and corresponds to hepatocellular carcinoma (HCC) in 90% of cases [2]. At diagnosis, 70% of HCC patients are classified as Barcelona Clinic of Liver Cancer (BCLC) B or C and only eligible for palliative treatments, mainly locoregional transarterial chemoembolization (TACE) or systemic therapies [3,4,5]. TACE is the treatment of choice for intermediate HCCs (BCLC B) with interesting objective tumor response rates around 50–55%, even though it is hampered by high rates of relapse. This procedure is associated with adverse events (AEs) such as fatigue, liver failure, or biliary infections [6]. This safety profile has to be imbalanced with the patients’ condition regarding their liver function, as well as the morphological characteristics of the tumor, including size and number of nodules and the presence or absence of macrovascular invasion. Even though it is well demonstrated that the best tumor response rates are observed in patients with preserved general status and liver function [7], other parameters such as the presence of significant comorbidities or the patient’s age could have a critical impact on the safety profile and efficacy of this technique. Age is a challenging feature given the significant heterogeneity of general conditions among individuals of the same age and the growing number of elderly patients in good clinical condition presenting with HCC. These actual demographic changes lead medical teams to constantly refine the boundaries of treatment indications [8]. Another critical issue is the lack of consensus regarding the definition of elderly patients. The World Health Organization (WHO) set the threshold at 65 years, which does not seem to be relevant anymore in current clinical practice. Moreover, therapeutic trials in oncology have been using different thresholds ranging from 65 to 80 years, but a majority used 70 years. Given that HCC incidence is constantly increasing and the worldwide population is aging, the management of HCC in elderly patients is becoming a major public health issue. However, the elderly population has been poorly studied in an HCC setting, with limited numbers of patients over 70 in phase 3 clinical trials and no trials dedicated to this specific population. This paradox is well illustrated by a recent meta-analysis on health-related quality of life in elderly cancer patients that pooled data from 25 EORTC randomized trials involving 6024 patients with only 9% of patients aged 70 years or more [9]. Thus, the aim of our study was to compare the safety profile and efficacy of TACE in the elderly, defined by an age of 70 years or more versus younger patients.

## 2. Patients and Methods

### 2.1. Patient Selection

We retrospectively included all patients who received a first TACE for HCC at the Grenoble-Alpes University Hospital from 1 January 2012 to 2 March 2017. Inclusion criteria were as follows: age > 18 years, HCC diagnosed by histology or based on non-invasive criteria [7], first trans-arterial chemoembolization, and available for pre- and post-therapeutic CT scans. Transarterial procedures performed for the acute bleeding of HCC were excluded.

### 2.2. Elderly Definition

Patients were divided into two groups as follows: elderly patients aged 70 years or more, in comparison with younger patients aged under 70 years.

### 2.3. TACE Procedure

TACE was performed following the standard local protocol as previously reported [10] and each indication was validated by a multidisciplinary board including at least a hepato-oncologist, an interventional radiologist, and a liver surgeon. The TACE technique could either use lipiodol or drug-eluting beads and anthracycline chemotherapy [11]. As recommended by European guidelines, TACE could be repeated 2 months after the first treatment in case of partial response on postoperative scan and preserved liver function and after validation in a multidisciplinary board [7].

### 2.4. Ethical Approval

Written consent was obtained for every patient before the transarterial procedures. The study complied with ethical standards and the Helsinki Declaration of 1975, as revised in 2008.

### 2.5. Data Collection and Safety Assessment

Patients’ data, including liver disease, HCC and TACE characteristics, comorbidities, as well as clinical and biological AEs or complications were collected from the patient’s electronic medical records.

AEs were qualified as early AEs when they occurred during the initial procedure hospitalization and delayed AEs when they occurred after discharge from the hospital and before the tumor response assessment performed usually 1–2 months after TACE.

We considered as serious adverse events (SAEs) the following elements:

- Migration to ECOG performance status of 3 or 4 after TACE;
- Liver decompensation after TACE, defined by the occurrence of ascites and/or hepatic encephalopathy (EH) (early and/or delayed) and/or a grade C Child–Pugh score after TACE;
- TACE-related death.

### 2.6. Efficacy Outcomes Analyses

Radiological response was assessed by comparing the baseline imaging and the imaging available at 1–2 months after the first session of trans-arterial treatment, as recommended [7]. Radiological response was assessed by radiologists with high expertise in HCC and TACE. Responses were classified as complete response, partial response, stable disease, and progressive disease as defined by the modified RECIST criteria [7]. All imaging was archived in a picture archiving and communication system (PACS).

Overall survival was defined as the time between the date of the first treatment and the date of death or the last recorded visit, with censure at the date of liver transplantation (LT) in transplanted patients.

## 3. Statistical Analysis

Descriptive analyses were used for the characterization of both groups of patients: the quantitative variables are presented as medians with interquartile ranges and the qualitative variables as frequencies (%).

The occurrence of AEs was compared between groups using exact Chi2 tests or Fisher tests for the comparison of qualitative variables and non-parametric Wilcoxon tests for quantitative variables. A univariate analysis based on a logistic model was performed to identify predictive factors of SAEs. Variables with a *p-*value < 0.2 in the univariate analysis were then included in a multivariate model. The collinearity between factors was tested and corrected, when necessary, by combining the two strongly correlated variables into one, or by keeping the variables with the least amount of missing data. Data with a *p-*value < 0.05 were considered statistically significant. Sensitivity analyses were carried out in order to not overlook an association between an older age (75 or 80 years) and the occurrence of SAEs. Survival charts were generated employing the Kaplan–Meier method, and survival rates between age groups were compared with a log-rank test. Statistical analyses were performed with SAS software, version 9.4 (Statistical Analysis System Institute, Cary, NC, USA).

## 4. Results

### 4.1. Description of the Population

During the study period, 271 patients received a first TACE for HCC at the Grenoble University Hospital, including 88 elderly patients and 183 in the younger patients’ group. The median follow-up of the patients was 2.3 (1.1–4.2) years.

Baseline patients’ characteristics are detailed in Table 1. The median age was 75 years in the group of elderly patients and 62 years in the group of younger patients (*p* < 0.01). Significantly more patients with a grade 2 ECOG performance status at baseline were observed in the elderly group (5.7% vs. 0.5%, *p* < 0.01). Elderly patients presented significantly more extrahepatic comorbidities such as arterial hypertension (63.6% vs. 50.3%, *p* = 0.04), cardiovascular diseases (29.5% vs. 17.5%, *p* = 0.02), and chronic kidney disease (CKD) (15.9% vs. 7.7%, *p* = 0.04). Elderly patients also more frequently had a past history of extrahepatic cancer (26.1% vs. 10.4%, *p* < 0.01).

Patients in the elderly group were less likely to have underlying cirrhosis (71.6% vs. 91.8%, *p* < 0.01) as well as less likely to have a history of liver decompensation (21.6% vs. 36.6%, *p* = 0.01). No significant differences in the Child–Pugh score, body mass index (BMI), or presence of comorbidities such as chronic respiratory diseases or diabetes were observed between groups.

The tumor burden was significantly greater in the elderly, with a median diameter of the largest nodule of 41 mm vs. 30 mm (*p* < 0.01) and a median total tumor volume of 80 mm vs. 58 mm (*p* < 0.01).

### 4.2. Safety Analyses

#### 4.2.1. Early Adverse Events

Early AEs are detailed in Table 2. No additional toxicity was observed in elderly patients concerning the vast majority of AEs, except post-puncture hematoma (4.5% vs. 0.5%, *p* = 0.02) and acute urinary retention (AUR) (9.1% vs. 2.7%, *p* = 0.02), which were more represented in the older group. No significant increase in the hospitalization length was observed (median = 4.5 days in elderly versus 5 days in younger patients; *p* = 0.60).

#### 4.2.2. Delayed Adverse Events

Delayed adverse events are detailed in Table 3. A significant increase in encephalopathy occurrence was observed in younger patients (5% vs. 0% in elderly; *p* = 0.04). Fatigue was more often reported in the elderly following TACE (40% vs. 24.4%, *p* < 0.01). There was no significant difference for the other clinical and biological parameters.

No differences were observed regarding treatment-related deaths (0 versus 4 deaths in elderly versus younger patients; *p* = 0.16), or re-admission rates before oncological reassessment (11.8% versus 18.3% in elderly versus younger patients; *p* = 0.18).

#### 4.2.3. Serious Adverse Events

SAEs, liver decompensations, serious deterioration in general condition, and treatment-related deaths are detailed in Table 3.

No statistically significant difference was observed between the two groups regarding SAEs with the occurrence of at least one SAE in 20.5% of elderly versus 21.3% in younger patients (*p* = 0.87). Additionally, there was no difference concerning treatment-related death rate and other safety outcomes.

## 5. Predictive Factors of Serious Adverse Events

The results of univariate and multivariate analyses for the study of the predictive factors of SAEs are indicated in Table 4. In the univariate analysis, the factors associated with the occurrence of SAEs are alcohol-related liver disease (*p* = 0.041), an ECOG grade greater than or equal to 1 (*p* = 0.0003), a history of liver decompensation (*p* < 0.0001), a MELD score greater than or equal to 9 (*p* = 0.0008), a Child–Pugh score greater than or equal to B7 (*p* < 0.0001), and a BCLC stage greater than or equal to B (*p* = 0.0364).

In contrast, there was no statistically significant association between an age of 70 years or older and the occurrence of SAEs (*p* = 0.87).

In the multivariate analysis, a Child score greater than or equal to B7 (*p* < 0.0001), an ECOG grade greater than or equal to 1 (*p* = 0.0019), and a MELD score greater than or equal to 9 (*p* = 0.0415) were associated with an increased risk of SAEs.

## 6. Sensitivity Analysis According to Age Threshold

There were 48 patients over 75 years old and 19 over 80 years old. The adverse events that occurred in these subgroups are listed in Supplementary Materials Tables S1 and S2.

In the group of patients 75 years old or more, we observed more acute kidney injury (14.6% vs. 3.1%, *p* < 0.01) and more acute urinary retention (16.7% vs. 2.2%, *p* < 0.01) during the initial hospitalization. Among delayed AEs, there was more fatigue (42.2% vs. 26.8%, *p* < 0.01) and more cardiac complications (6.7% vs. 1.4%, *p* = 0.04). The rate of post-TACE ascites was higher in patients over 75 years of age (17.8% vs. 8.2%, *p* = 0.05).

No significant difference was observed concerning other parameters such as death rate, length of hospitalization, or rate of SAEs.

In patients over 80 years of age, whereas comparable safety was observed during the initial hospitalization, more cardiac complications (16.7% vs. 1.2%, *p* < 0.01) and serious general status deterioration (11.1% vs. 2.0%, *p* = 0.02) were noted among delayed AEs.

An age over 75 was not a predictive factor of SAEs after multivariate analysis (OR 2.55 (0.898, 7.239), *p* = 0.0786) and an age over 80 was not evaluable due to a small number of patients.

## 7. Objective Tumor Response and Survival Outcomes

**Figure 1:**
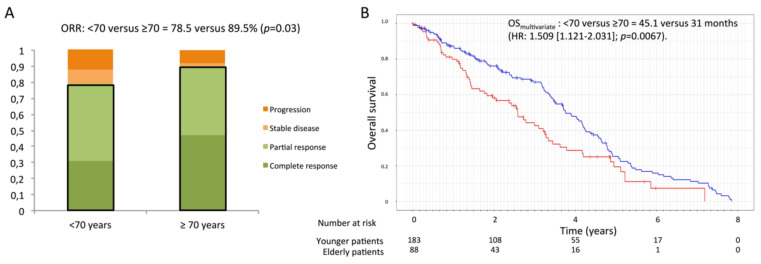
Efficacy outcomes of TACE according to age. (**A**) Objective tumor response on first imaging after TACE, divided into complete response, partial response, stable disease, and progression according to the Response Evaluation Criteria in Solid Tumors (mRECIST). The objective response rate (ORR), corresponding to the sum of complete and partial response rates, is represented by bold boxes. (**B**) Overall survival using the Kaplan–Meier method and compared using the log-rank test. Numbers at risk were reported under the *x*-axis.

Analyses of tumor response according to mRECIST at the first assessment with a median delay after TACE of 39 days (31;64), revealed a complete response in 37 (43.0%) elderly patients versus 84 (48.3%) patients under 70 and an objective tumor response (complete response + partial response) in 77 (89.5%) elderly patients versus 137 (78.7%) patients under 70 (*p* = 0.03) (Figure 1A). An objective tumor control (complete response + partial response + stable disease) was obtained in 79 (91.9%) elderly patients versus 152 (87.4%) patients under 70 (*p* = 0.28).

The median overall survival (OS) from TACE was 41.4 months in the overall cohort. After univariate analysis, the OS was 31.0 months in elderly patients versus 45.1 months in patients under 70 (*p* = 0.006). After multivariate analysis, age over 70 was associated with increased mortality (HR: 1.509, (1.121–2.031), *p* = 0.0067) (Figure 1B).

The overall survival after the censure of liver transplant events was 38.5 months in the global cohort, 31.2 in elderly patients, and 40.4 months in patients under 70 (*p* = 0.2556).

## 8. Discussion

The aim of this study was to describe the safety profile of TACE in patients with intermediate HCC over 70 years of age. To our knowledge, this is the largest study focusing on this topic, with 271 patients including 88 elderly patients. The elderly are under-represented in clinical research even though the worldwide population’s age is increasing, as well as cancer incidence, leading to a dramatic and steady increase in elderly patients with cancer in clinical practice. The scarcity of older patients in clinical trials limits physicians’ ability to make treatment decisions in this population as evidence of treatments’ efficacy and safety are lacking. Moreover, the question of safety is particularly essential in elderly patients due to their putative frailty and the risk of impairing autonomy with a major prejudice on quality of life. Although predictive factors of survival and tumor response to TACE have been extensively described, the determinants of SAE occurrence after TACE have been poorly studied.

Child–Pugh ≥ B7 was a strong predictive factor of serious adverse events (*p* < 0.0001), which is consistent with the existing literature and confirms how the appropriate selection of patients for TACE is paramount [12]. On the opposite, this study clearly demonstrated that an age over 70 years is not associated with an impaired safety profile. Moreover, patients over 75 and 80 years of age did not seem to experience increased toxicity. Age, even extreme, was not a predictive factor of SAE occurrence after TACE, as the elderly did not experience more SAEs, liver decompensations, or treatment-related deaths. Few AEs were increased in elderly patients, such as post-puncture hematoma (4.5% vs. 0.5%, *p* = 0.02) and acute urinary retention (AUR) (9.1% vs. 2.7%, *p* = 0.02). These AEs might easily be prevented by performing radial TACE, as this alternative allows a patient to immediately get up, limiting decubitus-related AEs. Besides, in our cohort, we observed a increase in tumor response in elderly (89.5% versus 78.7%; *p* = 0.03) without a significant difference on tumor control (91.9% versus 87.4%; *p* = 0.28). These data are not sufficient to conclude that elderly patients benefit from TACE more than younger patients, but it demonstrates that age does not negatively impact the anti-tumor efficacy of TACE. The overall survival was in favor of younger patients but these analyses were probably impacted by the age difference, which independently influences patients’ condition and survival, as well as liver transplantation, which was by definition contraindicated in elderly patients due to their age exceeding the limit of liver graft access. LT was performed in 31.7% of patients in the younger group. In this setting, TACE was performed as recommended to control tumor growth during the waiting time before LT, or in a down-staging strategy as a bridge to LT. None of the elderly patients were treated by TACE within an LT project as an age over 70 years is usually considered a contraindication to liver transplantation in our center. Thus, they were all treated with a palliative goal. This difference represents an important confusion bias regarding overall survival analyses. Overall survival after the censure of LT events may reduce the impact of LT on survival, and this outcome was not statistically different between groups (31.2 in elderly versus 40.4 months in patients under 70, *p* = 0.2556). Another interesting parameter to characterize TACE efficacy would have been progression-free survival (PFS). Nonetheless, due to the retrospective nature of this study, the date of progression was missing for too many patients to analyze PFS. Even though our study is more focused on safety, this constitutes a limit, as PFS would better illustrate survival outcomes after TACE independently of the patients’ age. However, these data strongly suggest that TACE may be considered in elderly patients with preserved clinical conditions if usual parameters such as tumor burden, liver function, and the severity of comorbidities are fulfilled.

Even though previous works reported comparable outcomes of TACE in the elderly, the number of patients was usually small, the definition of elderly patients was not consistent from one study to the other, and the control groups of younger patients were often lacking [13,14,15]. A large retrospective survey including several cohorts also showed that TACE can be effective and safe in the elderly [16]. However, the timeframe for inclusions extended over 20 years, exposing high risks of heterogeneity among patients and therapeutic procedures. Indeed, as TACE is not standardized, with multiples changes in procedures and routines in these last years, data from the 1980s and 1990s are probably not applicable to current-day patients and clinicians’ practices. This study provides a good opportunity to explore differences between elderly and younger patients, given a fairly large population from a unique center and a short inclusion period, limiting external bias such as inter-center heterogeneity or periprocedural care improvements over the years. Inversely, its monocentric character might reduce the generalizability of the results, which need to be confirmed in multicentric trials including elderly patients or prospective cohorts dedicated to this population with a more accurate assessment of general condition, including a measure of scores such as Activities of Daily Living, Instrumental Activities of Daily Living [17], or Geriatric-8 assessment [18] that were not performed in this study.

In conclusion, this study demonstrated no significant difference in safety profiles after a first transarterial chemoembolization for hepatocellular carcinoma between elderly and younger patients. Advanced age is not a predictive factor of serious adverse events and should not be considered as a limitation in itself in treatment decision-making. Moreover, anti-tumor efficacy was not negatively impacted by age. Our results also advocate for revisiting the upper limits of age in clinical trials in the HCC setting in order to gather the missing data pertaining to the safety and efficacy of treatment strategies in elderly patients searching for the optimal balance between cancer treatment and the maintenance of autonomy and quality of life.

## Figures and Tables

**Table 1:** Patients’ baseline characteristics.

Clinical and Biological Data	Age < 70	Age ≥ 70	Overall Cohort	*p*-Value
Male *	171 (93.4)	78 (88.6)	249 (91.9)	0.17
**Median Age (years) ^§^**	62 (56–65)	75 (71.5–79)	65 (58–71)	<0.01
**BMI (kg/m^2^) ^§^**	27.1 (24.2–29.7)	25.5 (24.2–28.8)	26.6 (24.2–29.4)	0.16
Presence of comorbidities *				
Arterial hypertension	92 (50.3)	56 (63.6)	148 (54.6)	0.04
Cardiovascular diseases	32 (17.5)	26 (29.5)	58 (21.4)	0.02
Smoking	138 (78.4)	60 (76)	128 (77.7)	<0.01
SAS	24 (13.1)	9 (10.2)	33 (12.1)	0.78
Chronic respiratory diseases	14 (7.7)	7 (8)	21 (7.7)	0.93
CKD	14 (7.7)	14 (15.9)	28 (10.3)	0.04
Diabetes	72 (39.3)	41 (46.6)	113 (41.7)	0.26
History of extrahepatic cancer *	19 (10.4)	23 (26.1)	42 (15.5)	<0.01
Chronic liver disease etiology *				
Alcohol	135 (73.8)	48 (54.5)	183 (67.5)	<0.01
NASH	59 (32.2)	41 (46.6)	100 (36.9)	0.02
Chronic viral hepatitis	66 (36.1)	14 (15.9)	80 (29.5)	<0.01
Other	6 (3.3)	8 (9.1)	14 (5.2)	0.04
Presence of cirrhosis *	168 (91.8)	63 (71.6)	231 (85.2)	<0.01
History of liver decompensation *	67 (36.6)	19 (21.6)	86 (31.7)	0.01
ECOG grade *				
0	147 (80.3)	57 (64.8)	204 (75.3)	<0.01
1	35 (19.1)	26 (29.5)	61 (22.5)	
2	1 (0.5)	5 (5.7)	6 (2.2)	
**MELD ^§^**	10 (8,12)	9 (8,10)	9 (8,11)	0.13
Child–Pugh class *				
A	121 (72.4)	55 (88.7)	176 (76.9)	0.10
B	42 (25.2)	7 (11.3)	49 (21.4)	
C	4 (2.4)	0 (0)	4 (1.7)	
Ascites *	16 (8.7)	4 (4.5)	20 (7.4)	0.22
Encephalopathy *	4 (2.2)	0 (0)	4 (1.5)	0.16
Albumin (g/l) ^§^	36 (31–41)	38 (34–41)	37 (32–41)	0.03
PT (%) ^§^	78 (64–87)	81 (72–93)	79 (67–89.5)	<0.01
Total bilirubin (mol/l) ^§^	16 (10–25.5)	12 (9–17)	15 (9–22)	<0.01
**HCC Characteristics**				
Single tumor *	44 (24)	23 (26.1)	67 (24.7)	0.71
Multiple tumors *	139 (76)	65 (73.9)	204 (75.3)	
Median number of tumors ^§^	3 (2–4)	3 (1.5–5)	3 (2–4)	0.93
Median size of largest nodule (mm) ^§^	30 (20–50)	41 (28–63)	35 (24–51)	<0.01
Median tumor volume (mm) ^§^	58 (36–85)	80 (44–110)	60.5 (41–90)	<0.01
Partial portal thrombosis *	13 (7.1)	4 (4.5)	17 (6.3)	0.42
Median AFP (ng/mL) ^§^	13.1 (5–55)	7.9 (4.3–80.6)	12.5 (5–56.2)	0.43
BCLC *				
A	71 (38.8)	23 (26.1)	94 (34.7)	0.06
B	97 (53)	50 (56.8)	147 (54.2)	
C	15 (8.2)	15 (17)	30 (11.1)	
**TACE Characteristics**				
Global *	106 (57.9)	50 (57.5)	156 (57.8)	0.94
Selective *	77 (42.1)	37 (42.5)	114 (42.2)	
Conventional TACE *	174 (95.6)	75 (86.2)	249 (92.6)	<0.01
Drug-eluting beads TACE *	8 (4.4)	12 (13.8)	20 (7.4)	
**Previous Treatments**				
Surgical resection *	23 (12.6)	12 (13.6)	35 (12.9)	0.81
Radiofrequency ablation *	18 (9.8)	14 (15.9)	32 (11.8)	0.15
**Post TACE Treatments**				
Median TACE number *	2 (1–3)	2 (1–3)	2 (1–3)	0.32
Liver transplantation *	58 (31.7)	0 (0)	58 (21.4)	<0.01
Surgical resection *	5 (2.7)	3 (3.4)	8 (3.0)	0.76
Radiofrequency ablation *	37 (20.2)	14 (15.9)	51 (18.8)	0.40
Systemic therapy *	43 (23.5)	28 (31.8)	71 (26.2)	0.14
SIRT *	9 (4.9)	3 (3.4)	12 (4.4)	0.57

Abbreviations: AFP: alpha-fetoprotein; BCLC: Barcelona Clinic Liver Cancer; BMI; body mass index; ECOG: Eastern Cooperative Oncology Group; CKD: chronic kidney disease; HCC: hepatocellular carcinoma, MELD: Model for End-stage Liver Disease; NASH: non-alcoholic steatohepatitis; PT: prothrombin time; SAS: sleep apnea syndrome; SIRT: selective internal radiation therapy; TACE: transarterial chemoembolization. * Numbers (Percentages); ^§^ Median (interquartile range).

**Table 2:** Early adverse events.

Early Adverse Events	<70	≥70	Overall Cohort	*p*-Value
Post-embolization syndrome *	117 (63.9)	57 (64.8)	174 (64.2)	0.89
Fever *	52 (28.4)	28 (31.8)	80 (29.5)	0.57
Abdominal pain *	117 (63.9)	56 (63.6)	173 (63.8)	0.96
Nausea/vomiting *	30 (16.4)	17 (19.3)	47 (17.3)	0.55
Fatigue *	29 (15.8)	14 (15.9)	43 (15.9)	0.99
Post-puncture hematoma *	1 (0.5)	4 (4.5)	5 (1.8)	0.02
Arterial complications *	8 (4.4)	2 (2.3)	10 (3.7)	0.39
Ischemic gastro-duodenal ulcer *	1 (0.5)	2 (2.3)	3 (1.1)	0.20
Ischemic cholecystitis *	11 (6)	6 (6.8)	17 (6.3)	0.80
Ischemic pancreatitis *	3 (1.6)	0 (0)	3 (1.1)	0.23
Venous thromboembolic events *	2 (1.1)	0 (0)	2 (0.7)	0.32
Diaphragmatic paralysis *	2 (1.1)	0 (0)	2 (0.7)	0.32
Bacterial infection *	8 (4.4)	7 (8)	15 (5.5)	0.23
AKI *	7 (3.8)	7 (8)	14 (5.2)	0.15
AUR *	5 (2.7)	8 (9.1)	13 (4.8)	0.02
Cardiac decompensation *	12 (6.6)	9 (10.2)	21 (7.7)	0.29
Diabetes decompensation *	17 (9.3)	12 (13.6)	29 (10.7)	0.28
Other metabolic disorders *	2 (1.1)	2 (2.3)	4 (1.5)	0.45
Ascites *	10 (5.5)	2 (2.3)	12 (4.4)	0.23
Encephalopathy *	18 (9.8)	8 (9.1)	26 (9.6)	0.85
Total bilirubin(mol/l) ^§^	30.5 (22, 51)	22 (16, 30)	27 (19, 44)	<0.01
PT (%) ^§^	63.5 (52, 76)	72 (61, 81)	66 (56, 78)	<0.01
Death *	1 (0.5)	1 (1.1)	2 (0.7)	0.60
Hospital stay (days) ^§^	5 (4, 7)	4.5 (4, 7)	5 (4, 7)	0.60
Extension of hospitalization *	97 (53)	44 (50)	141 (52)	0.64
Early global complications *	155 (84.7)	75 (85.2)	230 (84.9)	0.91

Abbreviations: AKI: acute kidney injury; AUR: acute urinary retention; PT: prothrombin time. * Numbers (Percentages); ^§^ Median (interquartile range).

**Table 3:** Delayed adverse events and overall adverse events.

Delayed Adverse Events, *n* (%)	<70	≥70	Overall Cohort	*p*-Value
Post-embolization syndrome *	13 (7.3)	8 (9.4)	21 (8)	0.55
Fever *	11 (6.1)	8 (9.4)	19 (7.2)	0.34
Abdominal pain *	25 (14)	10 (11.8)	35 (13.3)	0.62
Nausea/vomiting *	9 (5)	3 (3.5)	12 (4.5)	0.58
Fatigue *	44 (24.4)	34 (40)	78 (29.4)	0.01
Arterial complications *	10 (5.6)	2 (2.4)	12 (4.5)	0.24
Ischemic gastro-duodenal ulcer *	1 (0.6)	1 (1.2)	2 (0.8)	0.59
Ischemic cholecystitis *	6 (3.3)	0 (0)	6 (2.3)	0.09
Ischemic pancreatitis *	3 (1.7)	1 (1.2)	4 (1.5)	0.76
Venous thromboembolic events *	2 (1.1)	0 (0)	2 (0.8)	0.33
Diaphragmatic paralysis *	2 (1.1)	0 (0)	2 (0.8)	0.33
Bacterial infection *	8 (4.4)	7 (8.2)	15 (5.7)	0.21
AKI *	10 (5.6)	5 (5.9)	15 (5.7)	0.91
AUR *	2 (1.1)	0 (0)	2 (0.8)	0.33
Cardiac decompensation *	3 (1.7)	3 (3.5)	6 (2.3)	0.34
Diabetes decompensation *	4 (2.2)	0 (0)	4 (1.5)	0.17
Other metabolic disorders *	2 (1.1)	1 (1.2)	3 (1.1)	0.96
Ascites *	17 (9.4)	9 (10.6)	26 (9.8)	0.77
Encephalopathy *	9 (5)	0 (0)	9 (3.4)	0.04
Post-TACE Child–Pugh class *				0.83
A B C	104 (68.8)	35 (77.8)	139 (70.9)	
	35 (23.3)	7 (15.5)	42 (21.4)	
	12 (7.9)	3 (6.6)	15 (7.7)	
Post-TACE Child–Pugh score shift ^§^	0 (0; 1)	0 (0; 1)	0 (0; 1)	1
Death *	4 (2.2)	0 (0)	4 (1.5)	0.16
Rehospitalization *	33 (18.3)	10 (11.8)	43 (16.2)	0.18
Global delayed complications *	72 (40)	43 (50.6)	115 (43.4)	0.10
**Overall Adverse Events**				
Overall AEs (early + delayed) *	158 (86.3)	79 (89.8)	237 (87.5)	0.42
Overall serious adverse events *	39 (21.3)	18 (20.5)	57 (21)	0.87
Overall liver decompensations *	39 (21.3)	17 (19.3)	56 (20.7)	0.70
Overall serious deterioration in general condition *	5 (2.8)	2 (2.4)	7 (2.6)	0.84
Total deaths *	5 (2.7)	1 (1.1)	6 (2.2)	0.40

Abbreviations: AEs: adverse events; AKI: acute kidney injury; AUR: acute urinary retention; TACE: transarterial embolization. * Numbers (Percentages); ^§^ Median (interquartile range).

**Table 4:** Predictive factors of serious adverse events (univariate and multivariate analyses). *p*-values < 0.5 are reported in bold characters.

Univariate Analyses		OR	95% CI	*p*-Value
Age	Age ≥ 70	0.949	(0.507, 1.778)	0.8712
	Age ≥ 75	1.511	(0.738, 3.095)	0.2591
	Age ≥ 80	1.001	(0.319, 3.143)	0.9983
Gender	Women	1.114	(0.393, 3.162)	0.8389
BMI	BMI ≥ 26.5	0.763	(0.424, 1.372)	0.3666
Arterial hypertension		0.756	(0.421, 1.358)	0.3496
Cardiovascular diseases		0.736	(0.346, 1.565)	0.4254
Smoking		1.480	(0.673, 3.254)	0.3299
SAS		0.481	(0.162, 1.431)	0.1884
Chronic respiratory diseases		0.875	(0.282, 2.709)	0.8163
CKD		1.287	(0.518, 3.197)	0.5873
Diabetes		0.774	(0.424, 1.413)	0.4035
History of extrahepatic cancer		0.716	(0.3, 1.709)	0.4517
Cirrhosis		1.606	(0.639, 4.037)	0.3141
Alcohol		2.065	(1.03, 4.141)	0.0410
NASH		0.743	(0.399, 1.385)	0.3498
Chronic viral hepatitis		1.387	(0.746, 2.58)	0.3009
Other causes		2.190	(0.704, 6.813)	0.1757
History of liver decompensation		3.793	(2.066, 6.961)	<0.0001
ECOG grade	≥1	3.199	(1.717, 5.962)	0.0003
MELD	≥9	3.804	(1.745, 8.294)	0.0008
Child–Pugh	A	0.152	(0.076, 0.303)	<0.0001
Tumor morphology	Monofocal	1.729	(0.914, 3.271)	0.0923
Number of nodules	≥3	1.396	(0.768, 2.535)	0.2739
Size of largest nodule (mm)	≥35	0.981	(0.545, 1.768)	0.9504
Location	Unilobar	1.080	(0.602, 1.938)	0.7958
Partial portal thrombosis		1.619	(0.546, 4.799)	0.3852
AFP (ng/mL)	≥12.5	1.399	(0.76, 2.576)	0.2804
BCLC	≥B	2.061	(1.047, 4.059)	0.0364
Previous treatments		0.875	(0.43, 1.781)	0.7121
Area treated	Global	0.736	(0.408, 1.329)	0.3088
Type of TACE	c-TACE	1.532	(0.433, 5.426)	0.5082
Embolization of accessory tumor vasculature	Diaphragmatic arteries	0.638	(0.211, 1.927)	0.4253
	Mammary artery	1.000	(1, 1)	0.9998
	Other artery	1.673	(0.418, 6.693)	0.4669
Chemotherapy	Doxorubicin	2.921	(0.857, 9.959)	0.0867
**Multivariate Analyses**		**OR**	**95% CI**	***p*-Value**
Child–Pugh	≥B	5.034	(2.278, 11.126)	<0.0001
ECOG grade	≥1	3.556	(1.6, 7.903)	0.0019
MELD	≥9	2.450	(1.035, 5.798)	0.0415
Tumor morphology	Multifocal	0.423	(0.186, 0.96)	0.0396
Chemotherapy	Idarubicin	0.177	(0.036,0.886)	0.0351

Abbreviations: AFP: alpha-fetoprotein; BCLC: Barcelona Clinic Liver Cancer; BMI; body mass index; ECOG: Eastern Cooperative Oncology Group; CKD: chronic kidney disease; MELD: Model for End-stage Liver Disease; NASH: non-alcoholic steatohepatitis; PT: prothrombin time; SAS: sleep apnea syndrome; SIRT: selective internal radiation therapy; TACE: transarterial chemoembolization.

## Data Availability

Data presented in this study are available on request to the corresponding author. The data are not publicly available due to confidential data among data sets.

## Supplementary Materials

The following supporting information can be downloaded at: https://www.mdpi.com/article/10.3390/cancers14071634/s1, Supplementary Table S1: Early adverse events for patients over 75 or 80 years old; Supplementary Table S2: Late adverse events and overall serious adverse events in patients over 75 or 80 years old.

## References

[1] Bray F., Ferlay J., Soerjomataram I., Siegel R.L., Torre L.A., Jemal A. (2018). Global cancer statistics 2018: GLOBOCAN estimates of incidence and mortality worldwide for 36 cancers in 185 countries. CA Cancer J. Clin..

[2] Llovet J.M., Zucman-Rossi J., Pikarsky E., Sangro B., Schwartz M., Sherman M., Gores G. (2016). Hepatocellular carcinoma. Nat. Rev. Dis. Primers.

[3] Llovet J.M., Bruix J. (2003). Systematic review of randomized trials for unresectable hepatocellular carcinoma: Chemoembolization improves survival. Hepatology.

[4] Sieghart W., Hucke F., Peck-Radosavljevic M. (2015). Transarterial chemoembolization: Modalities, indication, and patient selection. J. Hepatol..

[5] Blanc J.F., Debaillon-Vesque A., Roth G., Barbare J.C., Baumann A.S., Boige V., Boudjema K., Bouattour M., Crehange G., Dauvois B. (2021). Hepatocellular carcinoma: French Intergroup Clinical Practice Guidelines for diagnosis, treatment and follow-up (SNFGE, FFCD, GERCOR, UNICANCER, SFCD, SFED, SFRO, AFEF, SIAD, SFR/FRI). Clin. Res. Hepatol. Gastroenterol..

[6] Lencioni R., De Baere T., Soulen M.C., Rilling W.S., Geschwind J.-F.H. (2016). Lipiodol transarterial chemoembolization for hepatocellular carcinoma: A systematic review of efficacy and safety data. Hepatology.

[7] European Association for The Study of The Liver (2018). EASL Clinical Practice Guidelines: Management of hepatocellular carcinoma. J. Hepatol..

[8] Marosi C., Köller M. (2016). Challenge of cancer in the elderly. ESMO Open.

[9] Quinten C., Coens C., Ghislain I., Zikos E., Sprangers M.A., Ringash J., Martinelli F., Ediebah D.E., Maringwa J., Reeve B.B. (2015). The effects of age on health-related quality of life in cancer populations: A pooled analysis of randomized controlled trials using the European Organisation for Research and Treatment of Cancer (EORTC) QLQ-C30 involving 6024 cancer patients. Eur. J. Cancer.

[10] Roth G.S., Benhamou M., Teyssier Y., Seigneurin A., Abousalihac M., Sengel C., Seror O., Ghelfi J., Ganne-Carrié N., Blaise L. (2021). Comparison of Trans-Arterial Chemoembolization and Bland Embolization for the Treatment of Hepatocellular Carcinoma: A Propensity Score Analysis. Cancers.

[11] Roth G.S., Teyssier Y., Abousalihac M., Seigneurin A., Ghelfi J., Sengel C., Decaens T. (2020). Idarubicin vs doxorubicin in transarterial chemoembolization of intermediate stage hepatocellular carcinoma. World J. Gastroenterol..

[12] Granito A., Bolondi L. (2017). Non-transplant therapies for patients with hepatocellular carcinoma and Child-Pugh-Turcotte class B cirrhosis. Lancet Oncol..

[13] Nishikawa H., Kita R., Kimura T., Ohara Y., Takeda H., Sakamoto A., Saito S., Nishijima N., Nasu A., Komekado H. (2014). Transcatheter Arterial Chemoembolization for Intermediate-Stage Hepatocellular Carcinoma: Clinical Outcome and Safety in Elderly Patients. J. Cancer.

[14] Cohen M.J. (2013). Trans-arterial chemo-embolization is safe and effective for very elderly patients with hepatocellular carcinoma. World J. Gastroenterol..

[15] Cheng H.-M., Tanaka T., Nishiofuku H., Chanoki Y., Horiuchi K., Masada T., Tatsumoto S., Matsumoto T., Marugami N., Kichikawa K. (2019). Safety and Prognosis of Transarterial Chemoembolization for Octogenarians with Hepatocellular Carcinoma. Cardiovasc. Interv. Radiol..

[16] Cohen M.J., Levy I., Barak O., Bloom A.I., Fernández-Ruiz M., Di Maio M., Perrone F., Poon R.T., Shouval D., Yau T. (2014). Trans-arterial chemo-embolization is safe and effective for elderly advanced hepatocellular carcinoma patients: Results from an international database. Liver Int..

[17] Katz S. (1983). Assessing Self-maintenance: Activities of Daily Living, Mobility, and Instrumental Activities of Daily Living. J. Am. Geriatr. Soc..

[18] Bellera C.A., Rainfray M., Mathoulin-Pélissier S., Mertens C., Delva F., Fonck M., Soubeyran P.L. (2012). Screening older cancer patients: First evaluation of the G-8 geriatric screening tool. Ann. Oncol..

