# Supplementary material for: Safety and Efficacy of Transarterial Chemoembolization in Elderly Patients with Intermediate Hepatocellular Carcinoma
Source: Cancers (Basel). 2022 Mar 23;14(7):1634. doi: 10.3390/cancers14071634 (PMC8997035; doi:10.3390/cancers14071634)
Supplement: Supplementary file 1 [file cancers-14-01634-s001.zip › cancers-1630030-supplementary.pdf]

Supplementary Table S1.: Early adverse events for patients over 75 or 80 years old.

Early Adverse Events	<75	≥75	*p*-Value	<80	≥80	*p*-Value
**Post-embolization syndrome ***	143 (64.1)	31 (64.6)	0.95	161 (63.9)	13 (68.4)	0.69
**Fever ***	64 (28.7)	16 (33.3)	0.52	74 (29.4)	6 (31.6)	0.84
**Abdominal pain ***	145 (65)	28 (58.3)	0.38	161 (63.9)	12 (63.2)	0.95
**Nausea/vomiting ***	37 (16.6)	10 (20.8)	0.48	42 (16.7)	5 (26.3)	0.28
**Fatigue ***	32 (14.3)	11 (22.9)	0.14	36 (14.3)	7 (36.8)	<0.01
**Post-puncture hematoma ***	4 (1.8)	1 (2.1)	0.89	5 (2)	0 (0)	0.54
**Arterial complications ***	9 (4)	1 (2.1)	0.52	9 (3.6)	1 (5.3)	0.71
**Ischemic gastro-duodenal ulcer ***	2 (0.9)	1 (2.1)	0.48	2 (0.8)	1 (5.3)	0.07
**Ischemic cholecystitis ***	14 (6.3)	3 (6.3)	0.99	15 (6)	2 (10.5)	0.43
**Ischemic pancreatitis ***	3 (1.3)	0 (0)	0.42	3 (1.2)	0 (0)	0.63
**Venous thromboembolic events ***	2 (0.9)	0 (0)	0.51	2 (0.8)	0 (0)	0.70
**Diaphragmatic paralysis ***	2 (0.9)	0 (0)	0.51	2 (0.8)	0 (0)	0.70
**Bacterial infection ***	10 (4.5)	5 (10.4)	0.10	14 (5.6)	1 (5.3)	0.96
**AKI ***	7 (3.1)	7 (14.6)	< 0.01	11 (4.4)	3 (15.8)	0.03
**AUR ***	5 (2.2)	8 (16.7)	< 0.01	10 (4)	3 (15.8)	0.02
**Heart complications ***	16 (7.2)	5 (10.4)	0.45	19 (7.5)	2 (10.5)	0.64
**Diabetes imbalance ***	22 (9.9)	7 (14.6)	0.34	26 (10.3)	3 (15.8)	0.46
**Other metabolic disorders ***	3 (1.3)	1 (2.1)	0.70	4 (1.6)	0 (0)	0.58
**Ascites ***	11 (4.9)	1 (2.1)	0.38	11 (4.4)	1 (5.3)	0.85
**HE ***	21 (9.4)	5 (10.4)	0.83	24 (9.5)	2 (10.5)	0.89
**Total bilirubin(µmol/l)** §	29 [21; 47]	21 [16; 27]	<0.01	28 [20; 45]	21 [14 ; 25]	<0.01
**PT (%)** §	64 [54; 76]	74 [63; 81]	<0.01	65 [55; 77]	76.5 [71; 87]	<0.01
**Death ***	1 (0.4)	1 (2.1)	0.23	1 (0.4)	1 (5.3)	0.02
**Hospital stay (days)** §	5 [4; 7]	5 [4; 7.5]	0.55	5 [4; 7]	5 [4; 8]	0.85
**Extension of hospitalization ***	113 (50.7)	28 (58.3)	0.34	131 (52)	10 (52.6)	0.96
**Early global complications ***	188 (84.3)	42 (87.5)	0.58	213 (84.5)	17 (89.5)	0.56

Abbreviations: AKI: acute kidney injury, AUR: acute urinary retention, HE: Hepatic encephalopathy, PT: prothrombin time. * Numbers (Percentages) § Median (interquartile range).

Supplementary Table S2. Late adverse events and overall serious adverse events in patients over 75 or 80 years old.

Late Adverse Events		<75	≥75	*p*-Value	<80	≥80	*p*-Value
**Post-embolization syndrome** *		20 (9.1)	1 (2.2)	0.12	21 (8.5)	0 (0)	0.20
**Fever** *		17 (7.8)	2 (4.4)	0.43	18 (7.3)	1 (5.6)	0.78
**Abdominal pain** *		32 (14.6)	3 (6.7)	0.15	34 (13.8)	1 (5.6)	0.32
**Nausea/vomiting** *		11 (5)	1 (2.2)	0.41	11 (4.5)	1 (5.6)	0.83
**Fatigue** *		59 (26.8)	19 (42.2)	0.04	69 (27.9)	9 (50)	0.05
**Arterial complications** *		11 (5)	1 (2.2)	0.41	11 (4.5)	1 (5.6)	0.83
**Ischemic gastro-duodenal ulcer** *		2 (0.9)	0 (0)	0.52	2 (0.8)	0 (0)	0.70
**Ischemic cholecystitis** *		6 (2.7)	0 (0)	0.26	6 (2.4)	0 (0)	0.50
**Ischemic pancreatitis** *		4 (1.8)	0 (0)	0.36	4 (1.6)	0 (0)	0.59
**Venous thromboembolic events** *		2 (0.9)	0 (0)	0.52	2 (0.8)	0 (0)	0.70
**Diaphragmatic paralysis** *		2 (0.9)	0 (0)	0.52	2 (0.8)	0 (0)	0.70
**Bacterial infection** *		10 (4.5)	5 (11.1)	0.08	13 (5.3)	2 (11.1)	0.30
**AKI** *		11 (5)	4 (8.9)	0.30	13 (5.3)	2 (11.1)	0.30
**AUR** *		2 (0.9)	0 (0)	0.52	2 (0.8)	0 (0)	0.70
**Heart complications** *		3 (1.4)	3 (6.7)	0.03	3 (1.2)	3 (16.7)	< 0.01
**Diabetes imbalance** *		4 (1.8)	0 (0)	0.36	4 (1.6)	0 (0)	0.59
**Other metabolic disorders** *		2 (0.9)	1 (2.2)	0.45	2 (0.8)	1 (5.6)	0.07
**Ascites** *		18 (8.2)	8 (17.8)	0.05	25 (10.1)	1 (5.6)	0.53
**HE** *		9 (4.1)	0 (0)	0.17	9 (3.6)	0 (0)	0.41
**Total bilirubin(µmol/l)** §		16.4 [10; 26]	12 [9; 17]	0.01	15.5 [10; 25]	11 [8; 17]	0.04
**PT (%)** §		74 [63; 85.5]	80 [70; 91]	0.04	74 [63 ; 86]	86 [77.5; 93.5]	0.01
**Albumin (g/l)** §		35 [31; 40]	36 [30; 40]	0.97	35.5 [30.5; 40]	36 [29; 40]	0.90
**ECOG grade** *	0	153 (69.5)	20 (44.4)	<0.01	167 (67.6)	6 (33.3)	<0.01
	1	48 (21.8)	15 (33.3)		57 (23.1)	6 (33.3)	
	2	14 (6.4)	8 (17.8)		18 (7.3)	4 (22.2)	
	3	0 (0)	2 (4.4)		0 (0)	2 (11.1)	
	4	5 (2.3)	0 (0)		5 (2)	0 (0)	
**Child-Pugh** *	A	124 (70.8)	15 (71.5)	0.62	135 (70.6)	4 (80)	0.62
	B	38 (21.7)	4 (19.1)		41 (21.4)	1 (20)	
	C	13 (7.4)	2 (9.5)		15 (7.7)	0 (0)	
**Death** *		4 (1.8)	0 (0)	0.36	4 (1.6)	0 (0)	0.59
**Rehospitalization** *		35 (15.9)	8 (17.8)	0.76	39 (15.8)	4 (22.2)	0.47
**Global delayed complications** *		91 (41.4)	24 (53.3)	0.14	105 (42.5)	10 (55.6)	0.28
**Overall complications** *		192 (86.1)	45 (93.8)	0.15	220 (87.3)	17 (89.5)	0.78
**Overall liver decompensations** *		44 (19.7)	12 (25)	0.41	53 (21)	3 (15.8)	0.59
**Serious general deterioration** *		5 (2.3)	2 (4.4)	0.41	5 (2)	2 (11.1)	0.02
**Total deaths** *		5 (2.2)	1 (2.1)	0.95	5 (2)	1 (5.3)	0.35

Abbreviations: AKI: acute kidney injury, AUR: acute urinary retention, HE: Hepatic encephalopathy, ECOG: Eastern Cooperative Oncology Group, PT: prothrombin time.* Numbers (Percentages) § Median (interquartile range).
